# Development of Fluoroquinolone Resistance through Antibiotic Tolerance in Campylobacter jejuni

**DOI:** 10.1128/spectrum.01667-22

**Published:** 2022-09-06

**Authors:** Myungseo Park, Jinshil Kim, Jill Feinstein, Kevin S. Lang, Sangryeol Ryu, Byeonghwa Jeon

**Affiliations:** a Division of Environmental Health Sciences, School of Public Health, University of Minnesota, Saint Paul, Minnesota, USA; b Department of Food and Animal Biotechnology, Research Institute for Agriculture and Life Sciences, Seoul National University, Seoul, Republic of Korea; c Department of Agricultural Biotechnology, Seoul National University, Seoul, Republic of Korea; d Center for Food and Bioconvergence, Seoul National University, Seoul, Republic of Korea; e Department of Veterinary and Biomedical Sciences, College of Veterinary Medicine, University of Minnesota, Saint Paul, Minnesota, USA; USDA-ARS

**Keywords:** *Campylobacter jejuni*, antibiotic tolerance, fluoroquinolone resistance, oxidative stress

## Abstract

Antibiotic tolerance not only enables bacteria to survive acute antibiotic exposures but also provides bacteria with a window of time in which to develop antibiotic resistance. The increasing prevalence of Campylobacter jejuni isolates resistant to clinically important antibiotics, particularly fluoroquinolones (FQs), is a global public health concern. Currently, little is known about antibiotic tolerance and its effects on resistance development in C. jejuni. Here, we show that exposure to ciprofloxacin or tetracycline at concentrations 10 and 100 times higher than the MIC induces antibiotic tolerance in C. jejuni, whereas gentamicin or erythromycin treatment causes cell death. Interestingly, FQ resistance rapidly develops in C. jejuni after tolerance induction by ciprofloxacin and tetracycline. Furthermore, after tolerance is induced, alkyl hydroperoxide reductase (AhpC) plays a critical role in reducing FQ resistance development by alleviating oxidative stress. Together, these results demonstrate that exposure of C. jejuni to antibiotics can induce antibiotic tolerance and that FQ-resistant (FQ^R^) C. jejuni clones rapidly emerge after tolerance induction. This study elucidates the mechanisms underlying the high prevalence of FQ^R^ C. jejuni and provides insights into the effects of antibiotic tolerance on resistance development.

**IMPORTANCE** Antibiotic tolerance compromises the efficacy of antibiotic treatment by extending bacterial survival and facilitating the development of mutations associated with antibiotic resistance. Despite growing public health concerns about antibiotic resistance in C. jejuni, antibiotic tolerance has not yet been investigated in this important zoonotic pathogen. Here, our results show that exposure of C. jejuni to ciprofloxacin or tetracycline leads to antibiotic tolerance development, which subsequently facilitates the emergence of FQ^R^ C. jejuni. Importantly, these antibiotics are commonly used in animal agriculture. Moreover, our study suggests that the use of non-FQ drugs in animal agriculture promotes FQ resistance development, which is crucial because antibiotic-resistant C. jejuni is primarily transmitted from animals to humans. Overall, these findings increase our understanding of the mechanisms of resistance development through the induction of antibiotic tolerance.

## INTRODUCTION

Antibiotic tolerance refers to the ability of an entire population of susceptible bacteria to withstand antibiotic treatment for prolonged periods of time (1). This is explicitly different from persistence, which occurs in a subpopulation of bacterial clones (2). Whether achieved through tolerance or persistence, extended survival of pathogenic bacteria under antibiotic treatment can cause prolonged and/or recurrent infections, resulting in adverse clinical outcomes and treatment failure (1, 2). Furthermore, the extended survival of tolerant and persistent bacteria under antibiotic treatment can provide a window of time for antibiotic-resistant bacteria to emerge (3, 4).

Campylobacter spp., particularly Campylobacter jejuni, is a leading bacterial cause of gastroenteritis, accounting for 400 to 500 million cases of diarrhea worldwide per year (5). C. jejuni colonizes the gastrointestinal tracts of a wide range of animals and is transmitted to humans mainly through foodborne routes (6). In addition, the increasing prevalence of Campylobacter isolates resistant to clinically important antibiotics is a serious public health concern. Importantly, the prevalence of fluoroquinolone-resistant (FQ^R^) Campylobacter is increasing at an alarming rate and has significantly compromised the efficacy of this critically important antibiotic class (7). Approximately 28.5% of campylobacteriosis cases in the United States are associated with FQ resistance (8), leading to adverse patient outcomes (9, 10). Other countries have a much higher prevalence of FQ^R^ Campylobacter, for example, 76% in Italy (11), 87% in China (12), and 89% in Thailand (13). The World Health Organization (WHO) classifies FQ^R^ Campylobacter as one of the high-priority pathogens for which new antimicrobials should be developed (14).

Despite public health concerns about antibiotic resistance in Campylobacter, little is known about whether Campylobacter can develop antibiotic tolerance and how tolerance may affect the development of antibiotic resistance. In this study, we demonstrate that C. jejuni develops antibiotic tolerance by exposure to high concentrations of ciprofloxacin (an FQ drug) or tetracycline. Importantly, FQ resistance is rapidly developed through antibiotic tolerance induced by FQs and non-FQ antibiotics. Our results suggest that antibiotic tolerance plays an important role in the development of FQ resistance in C. jejuni.

## RESULTS

### Tolerance induction in C. jejuni by exposure to high concentrations of antibiotics.

We hypothesized that C. jejuni develops tolerance during antibiotic exposure. To test this hypothesis, we utilized several different antibiotics: ciprofloxacin, erythromycin, tetracycline, and gentamicin. These were chosen because C. jejuni is likely to be exposed to these antibiotics during treatment of human campylobacteriosis or in livestock production (15–18). FQs (e.g., ciprofloxacin) are the most commonly used oral antibiotic for empirical treatment of gastroenteritis (15), erythromycin is the drug of choice for treating campylobacteriosis, and gentamicin and tetracyclines are alternative drugs for treating systemic infections with Campylobacter (16). In addition, tetracyclines are widely used for food-producing animals, such as cattle, one of the major natural hosts of C. jejuni (17–19). To evaluate bacterial survival during antibiotic treatment, we conducted time-kill assays by treating C. jejuni cultures with these antibiotics at concentrations 10 and 100 times higher than the MIC and measuring the viability of C. jejuni after washing out the drug.

**FIG 1:**
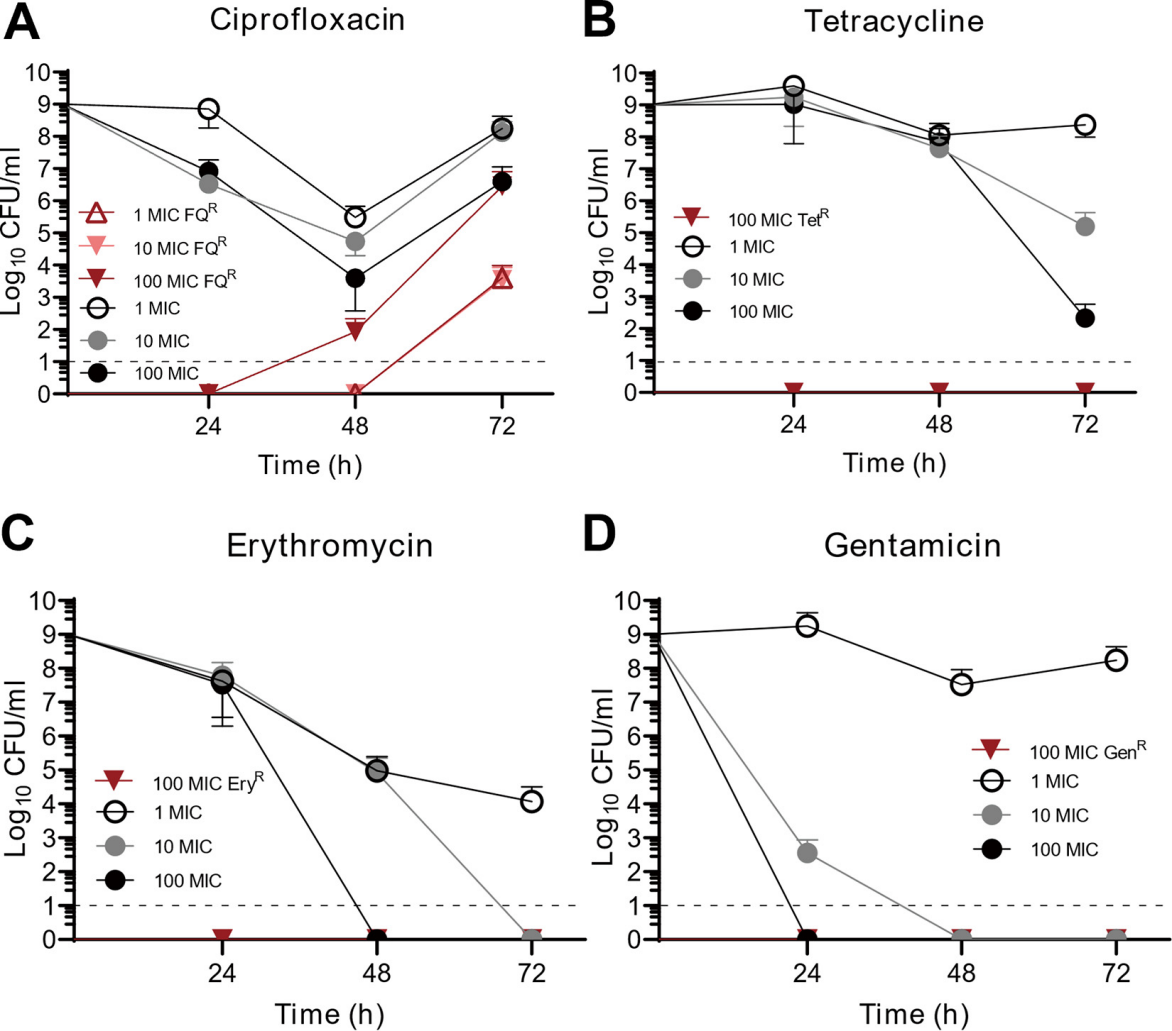
Induction of antibiotic tolerance in C. jejuni by exposure to high concentrations of ciprofloxacin (A), tetracycline (B), erythromycin (C), and gentamicin (D). The results show the means and standard deviations of the results of three independent experiments. Black lines indicate the total C. jejuni levels, and red lines show the levels of C. jejuni resistant to the antibiotic used for the treatment. Antibiotic concentrations were calculated based on the MICs of ciprofloxacin, tetracycline, erythromycin, and gentamicin in C. jejuni NCTC 11168, which were 0.063 μg/mL, 0.031 μg/mL, 1 μg/mL, and 0.5 μg/mL, respectively. The dotted lines indicate the limits of detection. Tet^R^, tetracycline resistant; Ery^R^, erythromycin resistant; Gen^R^, gentamicin resistant.

We found that C. jejuni survived treatment with ciprofloxacin or tetracycline (Fig. 1). Conversely, treatment with either gentamicin or erythromycin led to bacterial killing (Fig. 1). Translation inhibitors, such as tetracycline and erythromycin, are generally considered bacteriostatic but exhibited bactericidal activity when used at high concentrations (Fig. 1B and C). Resistance, tolerance, and persistence are differentiated primarily based on the kinetics of time-kill assays (1, 2, 20). Resistant bacteria survive and grow in the presence of an antibiotic. In contrast, tolerant cells can survive but not replicate in the presence of an antibiotic (1, 2, 20). Tolerance applies to an entire bacterial population, whereas persistence, or heterotolerance, is used to describe a tolerant subpopulation of cells (1, 2, 20, 21). Thus, in a time-kill assay, tolerant bacteria survive for long periods of time, compared to susceptible bacteria. However, persistence shows an initial killing pattern similar to that of susceptible bacteria, followed by a long survival duration, which generates a biphasic killing curve (1, 2, 20, 22). Our results show a pattern of delayed bacterial killing by these antibiotics, which is indicative of tolerance and not persistence (Fig. 1). Interestingly, treatment with high concentrations of ciprofloxacin did not lead to immediate bacterial killing. C. jejuni growth was initially inhibited but resumed after 48 h due to the emergence of FQ^R^ C. jejuni (Fig. 1A). These results show that tolerance can be induced in C. jejuni by exposure to high concentrations of ciprofloxacin or tetracycline, and FQ resistance rapidly develops during the window of time provided by antibiotic tolerance.

### Increases in oxidative stress by exposure to high concentrations of antibiotics in C. jejuni.

**FIG 2:**
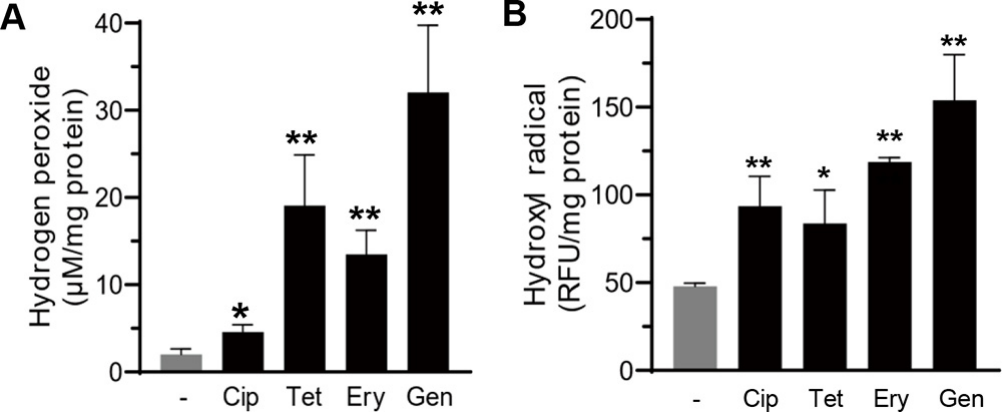
Formation of hydrogen peroxide (A) and hydroxyl radicals (B) by antibiotic exposure in C. jejuni. The levels of hydrogen peroxide and hydroxyl radicals were measured after exposure to 10× MIC of ciprofloxacin (Cip; 0.63 μg/mL), tetracycline (Tet; 0.31 μg/mL), erythromycin (Ery; 10 μg/mL), and gentamicin (Gen; 5 μg/mL) for 24 h. The results show the means and standard deviations of an experiment with three samples. The experiments were repeated three times and produced similar results. Statistical analysis was conducted using Student’s *t* test in comparison with an untreated control. RFU, relative fluorescence units; *, *P < *0.05; **, *P < *0.01.

Since oxidative stress is a general mechanism for bacterial lethality by bactericidal antibiotics (23), we hypothesized that C. jejuni must overcome increased oxidative stress after acute antibiotic exposure to maintain tolerance. To test the hypothesis, we measured hydrogen peroxide and hydroxyl radicals in C. jejuni during exposure to high levels of antibiotics. As predicted, the levels of hydrogen peroxide and hydroxyl radicals were significantly elevated after antibiotic treatment (Fig. 2). Notably, hydroxy radical levels were significantly increased by treatment with 10× MIC of erythromycin or gentamicin (Fig. 2B), which likely accounts for the rapid killing of C. jejuni by these antibiotics (Fig. 1C and D). Compared to these antibiotics, treatment with ciprofloxacin or tetracycline induced the formation of hydroxyl radicals at lower levels (Fig. 2). These results show that the level of hydroxyl radical formation correlates with whether antibiotic treatment leads to bacterial killing or induces antibiotic tolerance in C. jejuni.

### FQ resistance development by exposure to tolerance-inducing antibiotics in C. jejuni.

**FIG 3:**
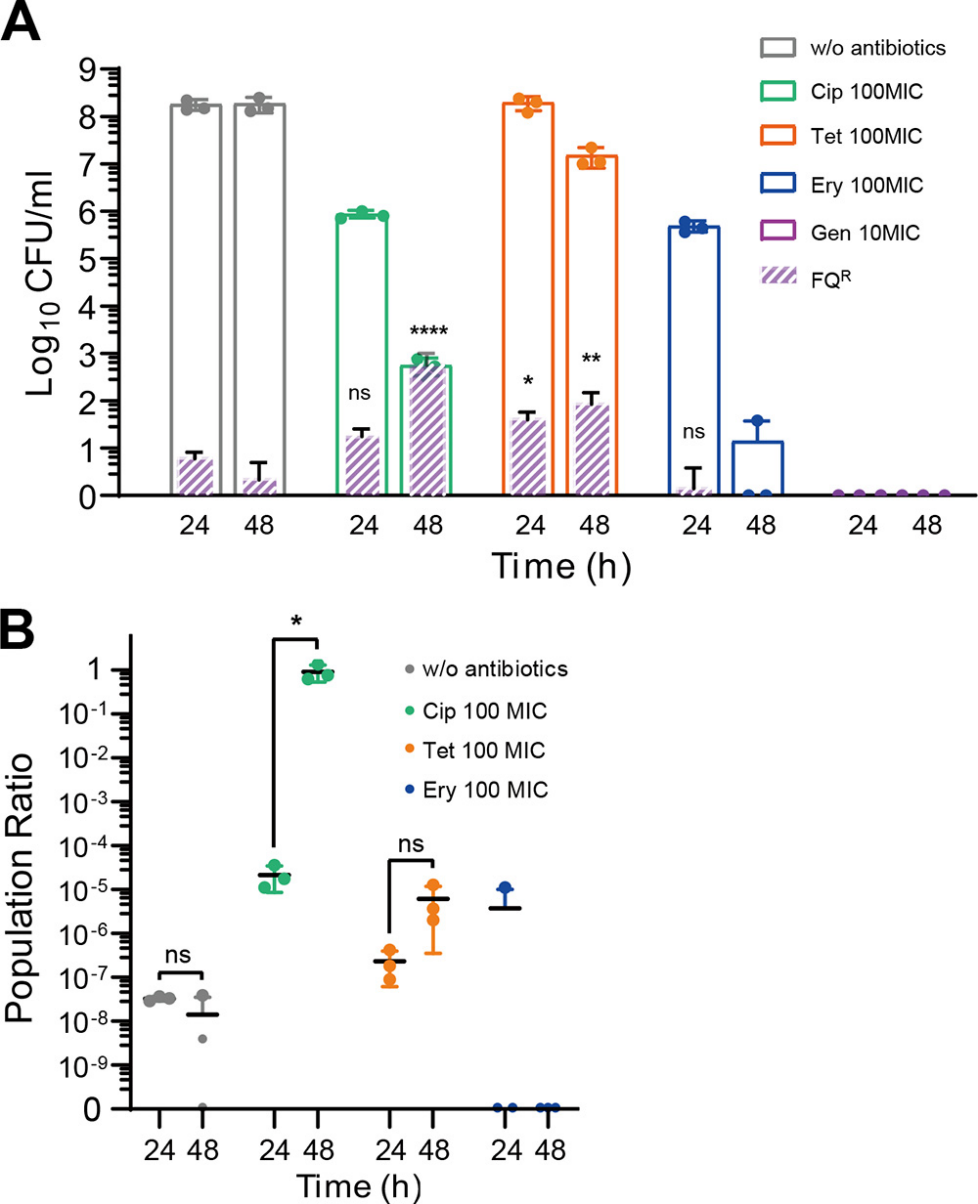
Development of fluoroquinolone (FQ) resistance during antibiotic tolerance in C. jejuni. (A) The emergence of FQ-resistant (FQ^R^) C. jejuni cells under treatment with 100× MIC of ciprofloxacin (Cip; 6.3 μg/mL), tetracycline (Tet; 3.1 μg/mL), and erythromycin (Ery; 100 μg/mL) and 10× MIC of gentamicin (Gen; 5 μg/mL). Gen was treated at a lower concentration due to its strong bactericidal activity. The results show the means and standard deviations of the levels of the total C. jejuni population (empty bars) and the FQ^R^ C. jejuni population (patterned filled bars) of the results from three independent experiments. Statistical analysis was performed using Student’s *t* test in comparison with an untreated control at the same sampling time. ns, nonsignificant; *, *P < *0.05; **, *P < *0.01; ****, *P < *0.0001. (B) Enrichment of FQ^R^ C. jejuni during antibiotic tolerance. The results show the ratio of FQ^R^ C. jejuni to the total C. jejuni. Statistical analysis was conducted using Student’s *t* test. ns, nonsignificant; *, *P < *0.05.

Oxidative stress during antibiotic treatment leads to increased DNA damage and subsequent mutations (23). Since antibiotic treatment increased hydroxyl radical formation in C. jejuni, we hypothesized that the frequency of FQ resistance mutations would increase. To test this hypothesis, we exposed C. jejuni to 100× MIC ciprofloxacin, 100× MIC tetracycline, 100× MIC erythromycin, and 10× MIC gentamicin. Interestingly, FQ^R^ C. jejuni clones rapidly emerged after exposure to tolerance-inducing antibiotics (Fig. 3A). Remarkably, the number of FQ^R^ cells arising during tetracycline treatment was significantly higher than that in a control without antibiotic treatment (Fig. 3A). The ratio of FQ^R^ to total C. jejuni was significantly increased in the presence of ciprofloxacin over 48 h (Fig. 3B). This suggests that FQ^R^ C. jejuni populations are enriched in the presence of ciprofloxacin during antibiotic tolerance. The emergence of FQ^R^ C. jejuni was not consistently observed in the presence of antibiotics with strong bactericidal activity. Gentamycin treatment rapidly killed C. jejuni, and erythromycin treatment induced the emergence of FQ^R^ C. jejuni isolates sporadically (one in three experiments) after 24 h but not after 48 h, likely due to bacterial killing by erythromycin (Fig. 3B).

FQ^R^ C. jejuni clones can emerge by spontaneous DNA mutations in the absence of antibiotics. Time-kill assays begin with large numbers of bacterial cells, and it is possible that FQ^R^ mutants exist in that initial population. We observed FQ^R^ mutants present in the control without antibiotic treatment (Fig. 3). However, antibiotic treatment further increased the levels of FQ^R^ mutants compared to the control, both in the presence of antibiotic selective pressure (i.e., ciprofloxacin treatment) and its absence (i.e., tetracycline) (Fig. 3). Ciprofloxacin treatment can enrich FQ^R^ C. jejuni clones that emerge by spontaneous DNA mutations prior to and during antibiotic exposure. In contrast, tetracycline treatment would not enrich FQ^R^ clones due to the lack of selection pressure. Despite this, we observed that FQ^R^ C. jejuni was more prevalent in the tetracycline-treated culture than in a nontreated control (Fig. 3). These results suggest that tetracycline treatment induces a higher rate of spontaneous mutations than that under untreated control conditions. Moreover, the emergence of FQ^R^ C. jejuni was more frequent during exposure to 100× MIC ciprofloxacin than to 10× MIC ciprofloxacin (Fig. 1A), indicating that the increased oxidative stress induced by a higher concentration of ciprofloxacin may promote the emergence of FQ^R^ C. jejuni. In addition, we also observed that tolerance-inducing antibiotics increased oxidative stress and FQ resistance development in other C. jejuni strains, including ATCC 33291 and ATCC 33560, which is used as a quality control strain for antimicrobial susceptibility testing (see Fig. S1 in the supplemental material). Altogether, these results suggest that FQ resistance rapidly develops in C. jejuni during antibiotic tolerance induced by FQs and non-FQ antibiotics.

### AhpC reduces FQ resistance development in C. jejuni during antibiotic tolerance.

**FIG 4:**
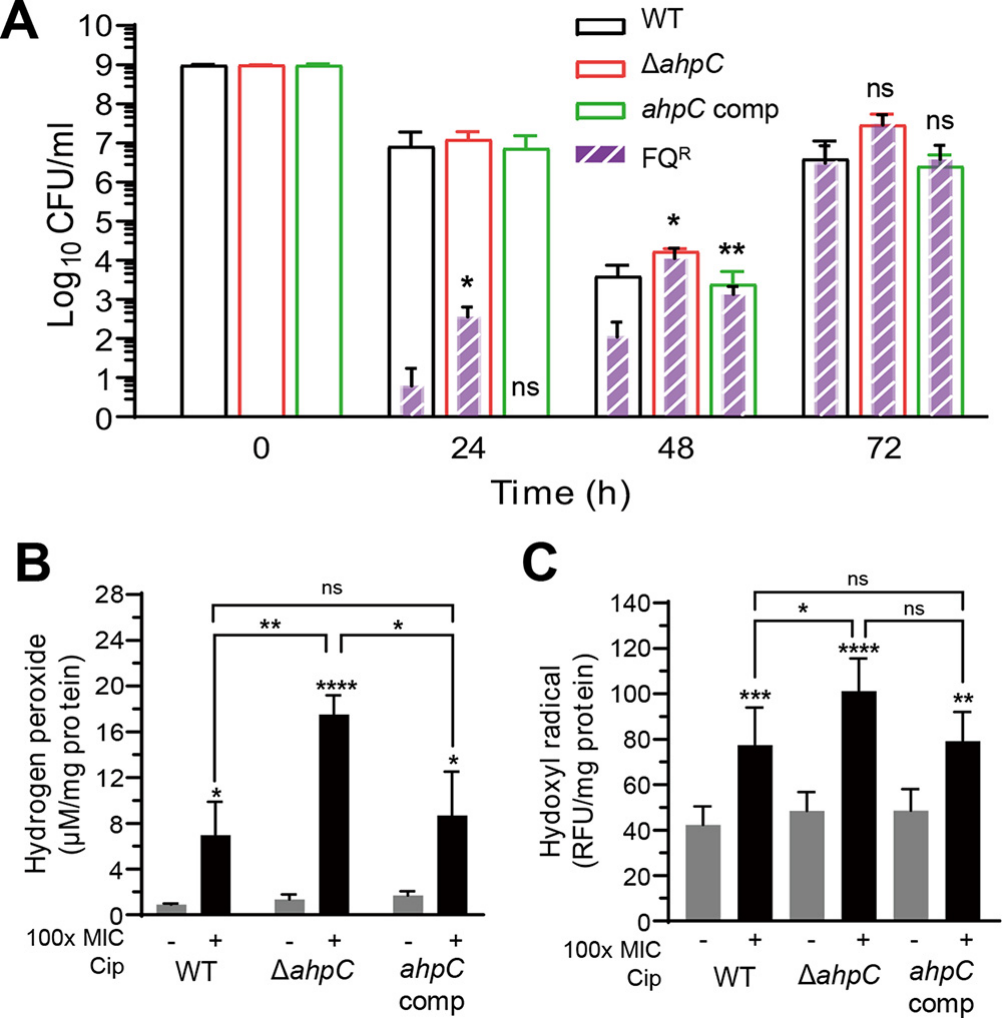
Enhanced development of fluoroquinolone (FQ) resistance in an Δ*ahpC* mutant during antibiotic tolerance induced by ciprofloxacin. (A) Significant increase in FQ resistance development in an Δ*ahpC* mutant during antibiotic tolerance induced by 100× MIC of ciprofloxacin (6.3 μg/mL). The results show the means and standard deviations of the levels of total C. jejuni (empty bars) and FQ^R^ C. jejuni (patterned filled bars) of the results from three independent experiments. Statistical analysis was conducted using Student’s *t* test in comparison with WT. ns, nonsignificant; *, *P < *0.05; **, *P < *0.01; *ahpC* comp, *ahpC*-complemented strain. Increased production of hydrogen peroxide (B) and hydroxyl radicals (C) in an Δ*ahpC* mutant. The + and – signs indicate the presence and absence of 100× MIC of ciprofloxacin (6.3 μg/mL), respectively. Statistical analysis was conducted using Student’s *t* test. ns, nonsignificant; *, *P < *0.05; **, *P < *0.01; ***, *P < *0.001; ****, *P < *0.0001.

Our results suggest that oxidative stress during antibiotic exposure leads to the emergence of FQ^R^ C. jejuni in antibiotic-tolerant cells. C. jejuni harbors a single copy of genes encoding alkyl hydroperoxide reductase (AhpC), catalase (KatA), and superoxide dismutase (SodB) (24), which are all involved in the detoxification of different reactive oxygen species. Using *ahpC*, *katA*, and *sodB* knockout mutants, we examined the contribution of each antioxidant enzyme to the prevention of FQ resistance development in C. jejuni during antibiotic tolerance. Remarkably, an Δ*ahpC* mutation significantly increased the frequency of FQ resistance development compared to that in the wild type (WT) (Fig. 4A). We observed no effect of Δ*katA* and Δ*sodB* mutations compared to WT (Fig. S2). In the Δ*ahpC* mutant, ciprofloxacin treatment markedly increased the accumulation of hydrogen peroxide (Fig. 4B), a substrate of AhpC, and hydroxyl radicals (Fig. 4C). These results suggest that increased oxidative stress facilitates the development of FQ resistance during antibiotic tolerance in C. jejuni and that AhpC reduces the development of FQ resistance by alleviating oxidative stress.

### Oxidative stress response regulators modulating *ahpC* transcription affect FQ resistance development during antibiotic tolerance in C. jejuni.

**FIG 5:**
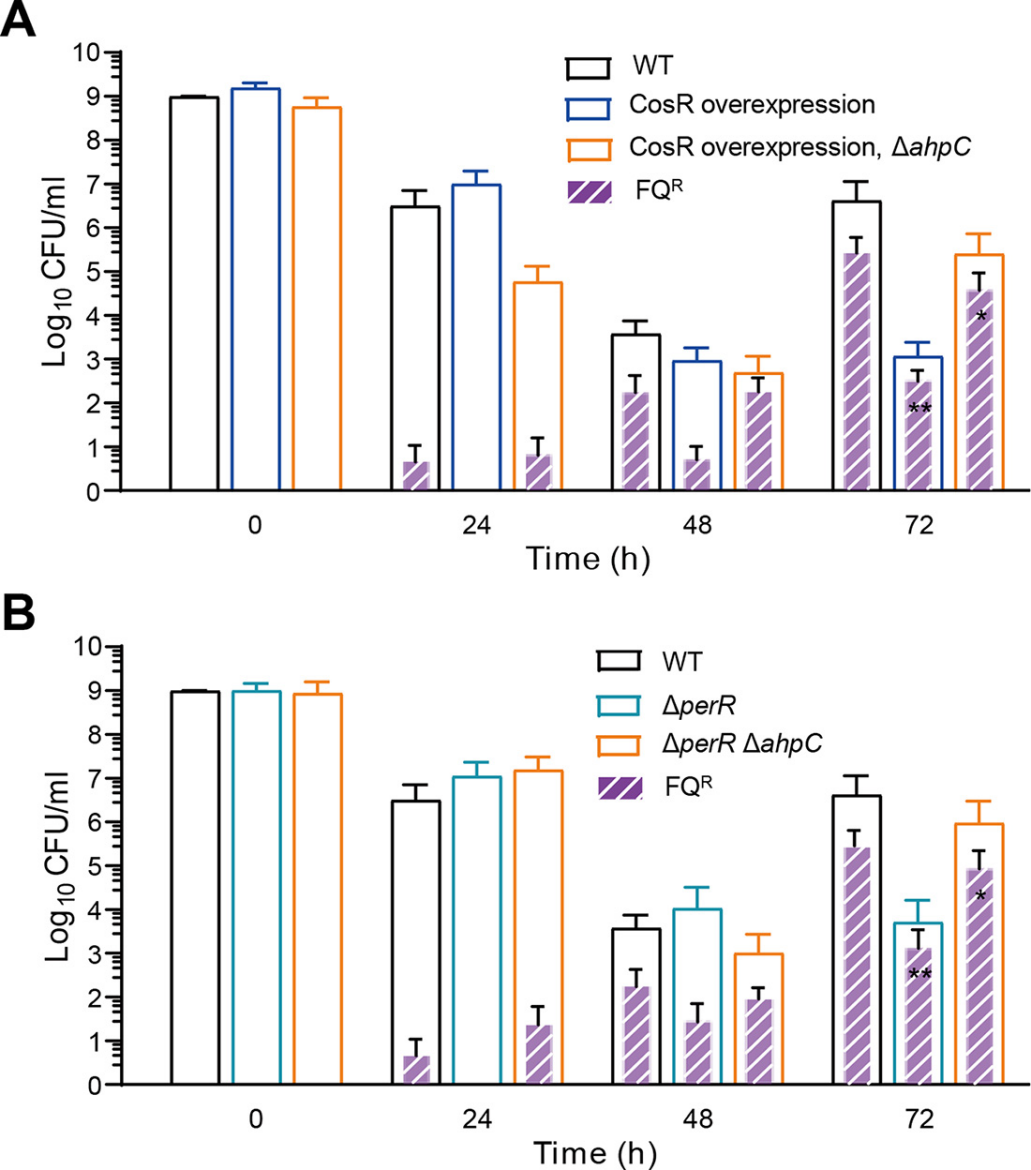
Effects of *ahpC* regulation on FQ resistance development in C. jejuni during antibiotic tolerance induced by ciprofloxacin. (A) FQ resistance development in a CosR overexpression strain and a CosR overexpression strain with an Δ*ahpC* mutation. (B) FQ resistance development in a Δ*perR* mutant and a Δ*perR* Δ*ahpC* mutant. The concentration of ciprofloxacin is 100× MIC (6.3 μg/mL). The results show the means and standard deviations of the levels of total C. jejuni (empty bars) and FQ^R^ C. jejuni (patterned filled bars) of the results from three independent experiments. Statistical analysis was conducted using two-way analysis of variance (ANOVA) followed by Dunnett’s multiple-comparison test. *, *P < *0.05; **, *P < *0.01.

To further confirm the role of AhpC in FQ resistance development during antibiotic tolerance, we used mutants defective in the regulation of oxidative stress responses. C. jejuni uses PerR (25) and CosR (26) to respond to oxidative stress. PerR is a repressor of *ahpC* transcription (25, 27). CosR is a response regulator that positively regulates *ahpC* transcription (26). Thus, a Δ*perR* mutation increases *ahpC* transcription by derepression, and CosR overexpression increases *ahpC* transcription by positive regulation. We measured the development of FQ resistance after inducing antibiotic tolerance in a CosR overexpression strain (Fig. 5A) and a Δ*perR* mutant (Fig. 5B). The frequency of FQ resistance development was substantially reduced in the Δ*perR* and CosR overexpression mutants. However, when *ahpC* was deleted in these mutants, the frequency of FQ resistance was increased to levels similar to those of the WT (Fig. 5), confirming that AhpC prevents FQ resistance development during antibiotic tolerance. Moreover, we observed that the level of *ahpC* transcription was significantly increased in C. jejuni during treatment with high concentrations of antibiotics (Fig. S3). This indicates that the antioxidation function of AhpC is required during antibiotic tolerance in C. jejuni. These results demonstrate that AhpC plays an important role in preventing the development of FQ resistance through tolerance induction in C. jejuni.

## DISCUSSION

**FIG 6:**
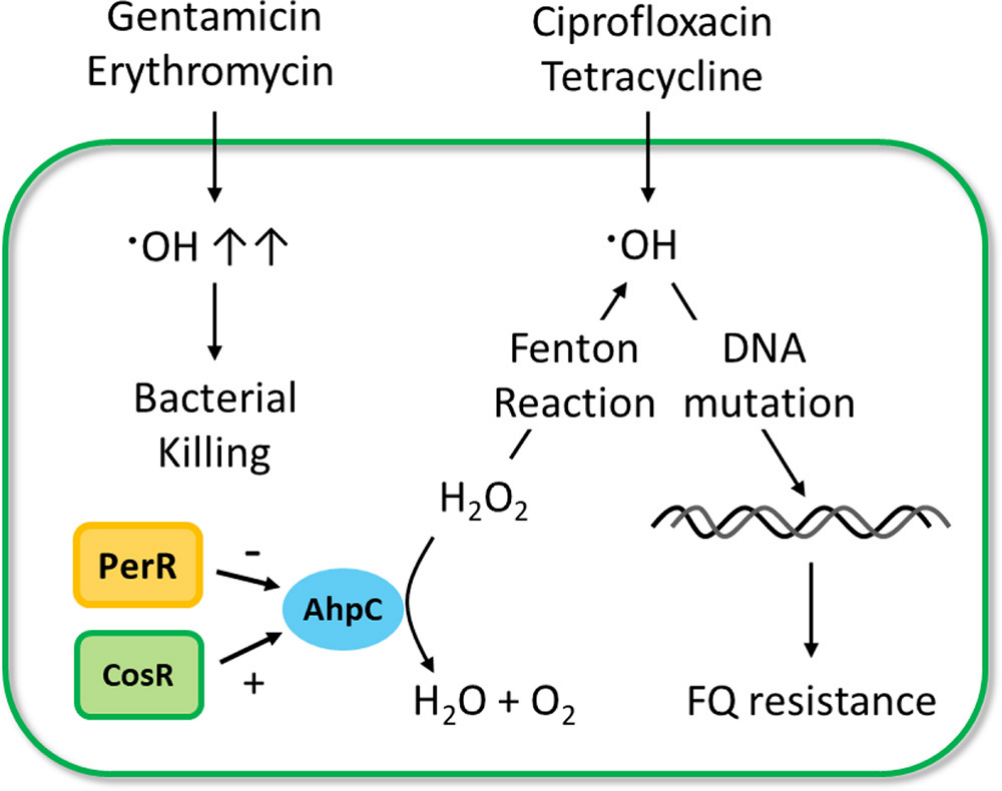
Schematic diagram of FQ resistance development through tolerance induction in C. jejuni. Exposure to high concentrations of gentamicin or erythromycin induces the formation of hydroxyl radicals at high levels, which can lead to cell death. Ciprofloxacin and tetracycline exposures do not generate lethal levels of hydroxyl radicals and cause DNA mutations, conferring FQ resistance. AhpC decreases the level of hydroxyl radicals by degrading hydrogen peroxide, which can be converted to hydroxyl radicals by the Fenton reaction. The *ahpC* transcription is negatively and positively regulated by PerR and CosR, respectively.

The data in this study show that antibiotic tolerance induced by exposure to FQs and non-FQ drugs promotes the development of FQ resistance in C. jejuni. Studies demonstrate that antibiotic tolerance or persistence induced by intermittent antibiotic exposures or drug combinations precedes the emergence of antibiotic resistance mutations in Escherichia coli and Staphylococcus aureus (3, 4). In E. coli, FQs induce the SOS response in persister cells and increase DNA mutations through the induction of error-prone DNA polymerases (28). However, C. jejuni lacks SOS response systems and error-prone DNA polymerases. Instead, our data demonstrate that AhpC plays a critical role in preventing FQ resistance development. AhpC is involved in the detoxification of organic peroxides and low physiological levels of hydrogen peroxide (29). Deletion of *ahpC* results in the accumulation of hydrogen peroxide and hydroxyl radicals (Fig. 4B and C), which increases the frequency of FQ resistance development during antibiotic tolerance (Fig. 4A). Hydrogen peroxide is generated by the accidental autoxidation of nonrespiratory flavoproteins (30, 31) and dismutation of superoxide (32) and is converted to hydroxyl radicals through the iron-catalyzed Fenton reaction (32). Thus, enzymatic degradation of hydrogen peroxide by AhpC can reduce oxidative DNA damage by toxic hydroxyl radicals and consequently decrease FQ resistance development during antibiotic tolerance in C. jejuni (Fig. 6). In E. coli, antibiotic treatment can lead to cell death without involving the formation of hydrogen peroxide under anaerobic conditions (33). It is not fully understood how reactive oxygen species (ROS) affect antimicrobial killing in C. jejuni. C. jejuni is an obligate microaerophile that grows optimally at low oxygen concentrations (34, 35). We observed that the level of hydrogen peroxide increased during exposure to high concentrations of antibiotics (Fig. 2A). Given these findings, antimicrobial lethality is likely to be associated with ROS generation during antibiotic treatment in C. jejuni, which may determine whether antibiotic treatment leads to bacterial killing or tolerance induction.

Exposure to high concentrations of antibiotics significantly increased the level of *ahpC* transcription (see Fig. S3 in the supplemental material). Interestingly, the level of *ahpC* transcription was higher in cells treated with tolerance-inducing antibiotics (i.e., ciprofloxacin and tetracycline) than in those treated with bacteria-killing antibiotics (i.e., erythromycin and gentamicin), although the bacteria-killing antibiotics produced more hydroxyl radicals than the tolerance-inducing antibiotics (Fig. 2C). There are two possibilities for this observation. First, the regulation of *ahpC* transcription may be critical for bacterial killing processes by antibiotics. Without significant upregulation of *ahpC*, C. jejuni may be more susceptible to antibiotics, which may result in bacterial killing. The other possibility is that due to the bactericidal activity of the bacteria-killing antibiotics, C. jejuni is already likely to undergo the process of bacterial death, which may slow down cellular responses to stress conditions.

FQ^R^ C. jejuni rapidly emerged after tolerance induction, but the change in the size of the FQ^R^ C. jejuni population was dependent on the antibiotic used for tolerance induction (Fig. 3). FQ^R^ C. jejuni appeared within 24 h under tetracycline treatment and remained at similar levels at 48 h (Fig. 3B). During ciprofloxacin treatment, the population of FQ^R^ C. jejuni significantly increased over time (Fig. 3B). This can be ascribed to selective pressure exerted by ciprofloxacin. Ciprofloxacin treatment killed FQ-susceptible C. jejuni cells over time, increasing the ratio of FQ^R^ to total C. jejuni (Fig. 3B). Growth was arrested in tolerant cells during antibiotic treatment; therefore, the increase in FQ^R^ C. jejuni cells may be due to an increased frequency in resistant cells during extended antibiotic exposure. It is also possible that selective enrichment increased the number of FQ^R^ C. jejuni cells over time, because the FQ^R^ C. jejuni populations did not increase significantly during tetracycline treatment (Fig. 3B). These data suggest that C. jejuni may not be in complete dormancy during antibiotic tolerance. Further studies are needed to understand the physiology of antibiotic-tolerant C. jejuni.

FQs are the oral antibiotic most commonly used to treat various bacterial infections (15, 36). FQs bind to DNA gyrase and topoisomerase IV, generate double-stranded breaks in DNA, and lead to bacterial cell death (37, 38); thus, DNA mutations leading to structural changes in topoisomerase IV and DNA gyrase are the major cause of FQ resistance (39, 40). However, genes encoding topoisomerase IV are absent in C. jejuni (16, 24), and mutations in DNA gyrase subunit B do not mediate FQ resistance in C. jejuni (41). Instead, C. jejuni develops FQ resistance by single-step point mutations in GyrA (DNA gyrase subunit A) (41, 42). FQ treatment of chickens colonized by C. jejuni does not completely eliminate C. jejuni; instead, FQ^R^ C. jejuni cells emerge within a few days of FQ treatment, increasing the number of C. jejuni cells (36). This is the same pattern of bacterial killing and FQ resistance development through antibiotic tolerance in our study (Fig. 1A), suggesting that antibiotic tolerance may contribute to FQ resistance development *in vivo*.

The high prevalence of FQ^R^ Campylobacter has been ascribed mainly to the use of FQs in agriculture, because C. jejuni is typically transmitted from food-producing animals to humans (16, 42, 43). Considerable efforts have been made to curb FQ resistance in Campylobacter by banning FQs in the poultry production and agriculture sectors in the United States (44). However, FQ resistance in Campylobacter continues to increase (45). This has been attributed to the fitness benefits of FQ resistance in C. jejuni. Once C. jejuni develops FQ resistance, FQ^R^ C. jejuni outcompetes FQ-sensitive C. jejuni in the intestines of chickens, the primary reservoir for C. jejuni, even in the absence of FQs (46). This results in a high prevalence of FQ^R^ C. jejuni in chickens. Our results show that the induction of antibiotic tolerance is an important mechanism for the rapid development of FQ resistance in C. jejuni, even when treated with non-FQ antibiotics.

It is noteworthy to mention that the interplay between resistance development and enrichment can collectively contribute to antibiotic resistance. Tetracyclines can stimulate the development of FQ resistance but do not enrich the population of FQ^R^ C. jejuni (Fig. 3). FQs play a major role in enriching the population of FQ^R^ C. jejuni by selectively inhibiting FQ-sensitive populations (Fig. 3). Tetracyclines were used in agriculture for years prior to the approval of FQs for control of early mortality in broiler chickens (47). However, significant increases in FQ^R^ Campylobacter isolates are mainly attributed to the agricultural use of FQs (48, 49). Based on the findings in this study, we can speculate that non-FQ drugs used in agriculture also facilitate the emergence of FQ^R^ C. jejuni more frequently than baseline spontaneous *gyrA* mutations and that the use of FQs in agriculture has resulted in substantial increases in the prevalence of FQ^R^ C. jejuni through selective enrichment. A recent study from Australia showed that a subpopulation of Campylobacter isolates from chickens exclusively resistant to FQs was detected, although Australia has never allowed for the use of FQs in chicken production (50). Compared to FQ resistance in other countries that ever used FQs in poultry production, e.g., 87% in China (12), 89% in Thailand (13), and 85.6% in South Korea (51), ciprofloxacin resistance was less frequently (14.8%) detected in Australia (50). Based on our findings, we speculate that non-FQ drugs used in agriculture may facilitate the emergence of subpopulations of FQ^R^ Campylobacter and that the absence of FQs in poultry production has not enriched the population of FQ^R^ Campylobacter in Australia. Future epidemiological studies investigating the association of the use of tetracyclines with FQ resistance are needed to validate this hypothesis.

Antibiotic tolerance induced by non-FQ drugs, particularly tetracycline, can facilitate the emergence of FQ^R^ C. jejuni. Tetracyclines are most widely used in agriculture, accounting for 66% and 31% of marketed agricultural antimicrobials in the United States (17) and the European Union (18), respectively. In the United States, the use of tetracyclines as growth promoters is no longer allowed, and they can be used only for therapeutic purposes. However, tetracyclines represent the largest volume of domestic sales of medically important antibiotics approved for use in food-producing animals, and about 3,948 tons of tetracyclines were sold and distributed for veterinary purposes in the United States in 2020 (17). When applied as medication in feed, more than 70% of tetracyclines are unmetabolized and excreted from animals into the environment (52). This increases the chances of bacterial exposure to high levels of tetracyclines, both inside and outside animals. Many countries widely use tetracyclines as in-feed antibiotics for growth promotion, disease prevention, and treatment (53). Although FQs have been banned in livestock production in many countries, the widespread use of tetracycline or other tolerance-inducing antibiotics in animals can contribute to the development of FQ resistance in C. jejuni.

In summary, our results demonstrate that antibiotic tolerance is crucial for developing FQ resistance by enabling C. jejuni to survive extensive exposure to antibiotics. Antibiotic treatment elevates oxidative stress, leading to DNA damage and a subsequent increase in mutation frequency. Among the antioxidant enzymes available in C. jejuni, AhpC plays a major role in preventing the development of FQ resistance. Overall, the findings in this study provide novel insights into the molecular mechanisms of antibiotic tolerance and resistance development and may explain the high prevalence of FQ resistance in C. jejuni.

## MATERIALS AND METHODS

### Bacterial strains and growth conditions.

C. jejuni NCTC 11168 was used as the WT in this study. The isogenic knockout mutants of Δ*ahpC* (54), Δ*katA* (55), Δ*sodB* (55), and Δ*perR* (56) and a CosR overexpression strain (26) were reported in our previous studies. A Δ*perR* Δ*ahpC* double mutant and a CosR overexpression strain with Δ*ahpC* were constructed by transforming the Δ*perR* mutant and the CosR overexpression strain with the genomic DNA of the Δ*ahpC* mutant using natural transformation (57). The C. jejuni strains were routinely grown at 42°C in Mueller-Hinton (MH) medium (Difco) under microaerobic conditions (5% O_2_, 10% CO_2_, and 85% N_2_).

### Antibiotic tolerance assay.

Overnight cultures of C. jejuni on MH agar plates were resuspended in 5 mL of MH broth in a 14-mL round-bottom tube (BD Falcon, USA) to an optical density at 600 nm (OD_600_) of 0.08. The bacterial suspension was incubated with shaking at 200 rpm under microaerobic conditions. After 7 h, antibiotic exposure was initiated by adding 1× MIC, 10× MIC, or 100× MIC of antibiotics (ciprofloxacin, tetracycline, erythromycin, and gentamicin). The concentrations were determined based on the MIC of the WT (i.e., C. jejuni NCTC 11168). The cultures were also treated with 1× MIC of antibiotics to compare the effects of a relatively lower concentration of antibiotics on tolerance. The MICs of ciprofloxacin, tetracycline, erythromycin, and gentamicin in C. jejuni NCTC 11168 were 0.063 μg/mL, 0.031 μg/mL, 1 μg/mL, and 0.5 μg/mL, respectively, as determined with a microdilution susceptibility test. Since these MICs show a wide concentration range, we could not use a fixed concentration for the four antibiotics. For instance, 1 μg/mL is equivalent to 15.9× MIC of ciprofloxacin, 32.3× MIC of tetracycline, 1× MIC of erythromycin, and 2× MIC of gentamicin in C. jejuni NCTC 11168. The growth of C. jejuni can be seriously affected by 1 μg/mL (i.e., 15.9× MIC) of ciprofloxacin but not by 1 μg/mL (i.e., 1× MIC) of erythromycin. Using the same fixed concentration for different antibiotics can generate different levels of antimicrobial activity. Thus, we decided the concentration of antibiotic treatment based on the MIC. For sampling, 1.2 mL of C. jejuni cultures were harvested and washed with fresh MH medium. After washing, C. jejuni cells were resuspended in 100 μL of MH broth and spread for enumeration onto MH agar plates and MH agar plates supplemented with 1 μg/mL ciprofloxacin. Colonies growing on MH agar plates supplemented with ciprofloxacin were randomly picked up and subjected to a broth microdilution susceptibility test to confirm resistance (58).

### Hydroxyl radical measurement.

C. jejuni cells were treated with high concentrations of antibiotics for 24 h as described above. C. jejuni cells were washed twice with phosphate-buffered saline (PBS) and concentrated 10-fold. The assay was conducted according to the manufacturer’s instructions (Molecular Probes HPF, Thermo Scientific, USA). Briefly, 100 μL of a sample was placed onto a 96-well plate (black opaque; Corning, USA). Hydroxyphenyl fluorescein (HPF) solution was diluted to a final concentration of 5 μM. The fluorescence at excitation/emission (ex/em) values of 530/590 nm was measured using a plate reader (Varioskan Flash; Thermo Fisher Scientific) with gentle shaking at 25°C. Measured fluorescence signals were normalized to protein concentrations determined using a Bradford assay.

### Hydrogen peroxide measurement.

The level of hydrogen peroxide formation under antibiotic treatment was measured with the Amplex Red hydrogen peroxide/peroxidase assay kit (Invitrogen, Thermo Fisher Scientific), according to the manufacturer’s protocol. C. jejuni cells were exposed to antibiotics for 24 h as described above. The samples were washed twice, concentrated 10-fold, and placed onto a 96-well plate (black opaque; Corning). The working solution (10 mM Amplex Red reagent, 10 U/mL Horseradish peroxidase stock solution, and reaction buffer) was added to each sample solution. The mixture was incubated at room temperature for 30 min, and fluorescence was detected at ex/em 530/590 nm using a plate reader (Varioskan Flash; Thermo Fisher Scientific). The hydrogen peroxide concentration was determined by comparing it with a standard curve prepared with known hydrogen peroxide concentrations and normalized to protein concentrations determined using a Bradford assay.

## References

[1] Brauner A, Fridman O, Gefen O, Balaban NQ. 2016. Distinguishing between resistance, tolerance and persistence to antibiotic treatment. Nat Rev Microbiol 14:320–330. doi:10.1038/nrmicro.2016.34. 27080241

[2] Balaban NQ, Helaine S, Lewis K, Ackermann M, Aldridge B, Andersson DI, Brynildsen MP, Bumann D, Camilli A, Collins JJ, Dehio C, Fortune S, Ghigo JM, Hardt WD, Harms A, Heinemann M, Hung DT, Jenal U, Levin BR, Michiels J, Storz G, Tan MW, Tenson T, Van Melderen L, Zinkernagel A. 2019. Definitions and guidelines for research on antibiotic persistence. Nat Rev Microbiol 17:441–448. doi:10.1038/s41579-019-0196-3. 30980069 PMC7136161

[3] Levin-Reisman I, Ronin I, Gefen O, Braniss I, Shoresh N, Balaban NQ. 2017. Antibiotic tolerance facilitates the evolution of resistance. Science 355:826–830. doi:10.1126/science.aaj2191. 28183996

[4] Liu J, Gefen O, Ronin I, Bar-Meir M, Balaban NQ. 2020. Effect of tolerance on the evolution of antibiotic resistance under drug combinations. Science 367:200–204. doi:10.1126/science.aay3041. 31919223

[5] Kirk MD, Pires SM, Black RE, Caipo M, Crump JA, Devleesschauwer B, Dopfer D, Fazil A, Fischer-Walker CL, Hald T, Hall AJ, Keddy KH, Lake RJ, Lanata CF, Torgerson PR, Havelaar AH, Angulo FJ. 2015. World Health Organization estimates of the global and regional disease burden of 22 foodborne bacterial, protozoal, and viral diseases, 2010: a data synthesis. PLoS Med 12:e1001921. doi:10.1371/journal.pmed.1001921. 26633831 PMC4668831

[6] Facciolà A, Riso R, Avventuroso E, Visalli G, Delia SA, Laganà P. 2017. *Campylobacter*: from microbiology to prevention. J Prev Med Hyg 58:E79–E92. 28900347 PMC5584092

[7] Shen Z, Wang Y, Zhang Q, Shen J, Aarestrup FM, Schwarz S, Shen J, Cavaco L. 2018. Antimicrobial resistance in *Campylobacter* spp. Microbiol Spectr 6:6.2.11. doi:10.1128/microbiolspec.ARBA-0013-2017. PMC11633568 29623873

[8] CDC. 2019. Antibiotic resistance threats in the United States. doi:10.15620/cdc:82532.

[9] Helms M, Simonsen J, Olsen KE, Molbak K. 2005. Adverse health events associated with antimicrobial drug resistance in *Campylobacter* species: a registry-based cohort study. J Infect Dis 191:1050–1055. doi:10.1086/428453. 15747238

[10] Engberg J, Neimann J, Nielsen EM, Aerestrup FM, Fussing V. 2004. Quinolone-resistant *Campylobacter* infections: risk factors and clinical consequences. Emerg Infect Dis 10:1056–1063. doi:10.3201/eid1006.030669. 15207057 PMC3323146

[11] Garcia-Fernandez A, Dionisi AM, Arena S, Iglesias-Torrens Y, Carattoli A, Luzzi I. 2018. Human *Campylobacter*iosis in Italy: emergence of multi-drug resistance to ciprofloxacin, tetracycline, and erythromycin. Front Microbiol 9:1906. doi:10.3389/fmicb.2018.01906. 30186251 PMC6113387

[12] Zhou J, Zhang M, Yang W, Fang Y, Wang G, Hou F. 2016. A seventeen-year observation of the antimicrobial susceptibility of clinical *Campylobacter jejuni* and the molecular mechanisms of erythromycin-resistant isolates in Beijing, China. Int J Infect Dis 42:28–33. doi:10.1016/j.ijid.2015.11.005. 26594011

[13] Mason CJ, Sornsakrin S, Seidman JC, Srijan A, Serichantalergs O, Thongsen N, Ellis MW, Ngauy V, Swierczewski BE, Bodhidatta L. 2017. Antibiotic resistance in *Campylobacter* and other diarrheal pathogens isolated from US military personnel deployed to Thailand in 2002–2004: a case–control study. Trop Dis Travel Med Vaccines 3:13. doi:10.1186/s40794-017-0056-y. 28883983 PMC5530911

[14] World Health Organization. 27 February 2017. WHO publishes list of bacteria for which new antibiotics are urgently needed. http://www.who.int/mediacentre/news/releases/2017/bacteria-antibiotics-needed/en/.

[15] Schierenberg A, Bruijning-Verhagen PCJ, van Delft S, Bonten MJM, de Wit NJ. 2019. Antibiotic treatment of gastroenteritis in primary care. J Antimicrob Chemother 74:207–213. doi:10.1093/jac/dky385. 30285243

[16] Luangtongkum T, Jeon B, Han J, Plummer P, Logue CM, Zhang Q. 2009. Antibiotic resistance in *Campylobacter*: emergence, transmission and persistence. Future Microbiol 4:189–200. doi:10.2217/17460913.4.2.189. 19257846 PMC2691575

[17] US FDA. 2021. 2020 summary report on antimicrobials sold or distributed for use in food-producing animals.

[18] European Medicines Agency. 2020. Sales of veterinary antimicrobial agents in 31 European countries in 2018. https://amcra.be/swfiles/files/ESVAC-report-2018.pdf.

[19] Stanley K, Jones K. 2003. Cattle and sheep farms as reservoirs of *Campylobacter*. J Appl Microbiol 94:104–113. doi:10.1046/j.1365-2672.94.s1.12.x. 12675942

[20] Westblade LF, Errington J, Dörr T. 2020. Antibiotic tolerance. PLoS Pathog 16:e1008892. doi:10.1371/journal.ppat.1008892. 33057409 PMC7561168

[21] Windels EM, Michiels JE, Van den Bergh B, Fauvart M, Michiels J, Epstein S, Rubin EJ. 2019. Antibiotics: combatting tolerance to stop resistance. mBio 10:e02095-19. doi:10.1128/mBio.02095-19. 31506315 PMC6737247

[22] Fisher RA, Gollan B, Helaine S. 2017. Persistent bacterial infections and persister cells. Nat Rev Microbiol 15:453–464. doi:10.1038/nrmicro.2017.42. 28529326

[23] Kohanski MA, Dwyer DJ, Hayete B, Lawrence CA, Collins JJ. 2007. A common mechanism of cellular death induced by bactericidal antibiotics. Cell 130:797–810. doi:10.1016/j.cell.2007.06.049. 17803904

[24] Parkhill J, Wren BW, Mungall K, Ketley JM, Churcher C, Basham D, Chillingworth T, Davies RM, Feltwell T, Holroyd S, Jagels K, Karlyshev AV, Moule S, Pallen MJ, Penn CW, Quail MA, Rajandream MA, Rutherford KM, van Vliet AH, Whitehead S, Barrell BG. 2000. The genome sequence of the food-borne pathogen *Campylobacter jejuni* reveals hypervariable sequences. Nature 403:665–668. doi:10.1038/35001088. 10688204

[25] van Vliet AH, Baillon ML, Penn CW, Ketley JM. 1999. *Campylobacter jejuni* contains two fur homologs: characterization of iron-responsive regulation of peroxide stress defense genes by the PerR repressor. J Bacteriol 181:6371–6376. doi:10.1128/JB.181.20.6371-6376.1999. 10515927 PMC103772

[26] Hwang S, Kim M, Ryu S, Jeon B. 2011. Regulation of oxidative stress response by CosR, an essential response regulator in *Campylobacter jejuni*. PLoS One 6:e22300. doi:10.1371/journal.pone.0022300. 21811584 PMC3139631

[27] Handley RA, Mulholland F, Reuter M, Ramachandran VK, Musk H, Clissold L, Le Brun NE, van Vliet AHM. 2015. PerR controls oxidative stress defence and aerotolerance but not motility-associated phenotypes of *Campylobacter jejuni*. Microbiology (Reading) 161:1524–1536. doi:10.1099/mic.0.000109. 25968890

[28] Barrett TC, Mok WWK, Murawski AM, Brynildsen MP. 2019. Enhanced antibiotic resistance development from fluoroquinolone persisters after a single exposure to antibiotic. Nat Commun 10:1177. doi:10.1038/s41467-019-09058-4. 30862812 PMC6414640

[29] Seaver LC, Imlay JA. 2001. Alkyl hydroperoxide reductase is the primary scavenger of endogenous hydrogen peroxide in *Escherichia coli*. J Bacteriol 183:7173–7181. doi:10.1128/JB.183.24.7173-7181.2001. 11717276 PMC95566

[30] Korshunov S, Imlay JA. 2010. Two sources of endogenous hydrogen peroxide in *Escherichia coli*. Mol Microbiol 75:1389–1401. doi:10.1111/j.1365-2958.2010.07059.x. 20149100 PMC3049997

[31] Seaver LC, Imlay JA. 2004. Are respiratory enzymes the primary sources of intracellular hydrogen peroxide? J Biol Chem 279:48742–48750. doi:10.1074/jbc.M408754200. 15361522

[32] Imlay JA. 2013. The molecular mechanisms and physiological consequences of oxidative stress: lessons from a model bacterium. Nat Rev Microbiol 11:443–454. doi:10.1038/nrmicro3032. 23712352 PMC4018742

[33] Liu Y, Imlay JA. 2013. Cell death from antibiotics without the involvement of reactive oxygen species. Science 339:1210–1213. doi:10.1126/science.1232751. 23471409 PMC3731989

[34] Sellars MJ, Hall SJ, Kelly DJ. 2002. Growth of *Campylobacter jejuni* supported by respiration of fumarate, nitrate, nitrite, trimethylamine-N-oxide, or dimethyl sulfoxide requires oxygen. J Bacteriol 184:4187–4196. doi:10.1128/JB.184.15.4187-4196.2002. 12107136 PMC135223

[35] Kaakoush NO, Miller WG, De Reuse H, Mendz GL. 2007. Oxygen requirement and tolerance of *Campylobacter jejuni*. Res Microbiol 158:644–650. doi:10.1016/j.resmic.2007.07.009. 17890061

[36] Andersson MI, MacGowan AP. 2003. Development of the quinolones. J Antimicrob Chemother 51 Suppl 1:1–11. doi:10.1093/jac/dkg212. 12702698

[37] Drlica K, Malik M, Kerns RJ, Zhao X. 2008. Quinolone-mediated bacterial death. Antimicrob Agents Chemother 52:385–392. doi:10.1128/AAC.01617-06. 17724149 PMC2224783

[38] Aldred KJ, Kerns RJ, Osheroff N. 2014. Mechanism of quinolone action and resistance. Biochemistry 53:1565–1574. doi:10.1021/bi5000564. 24576155 PMC3985860

[39] Hooper DC. 2001. Emerging mechanisms of fluoroquinolone resistance. Emerg Infect Dis 7:337–341. doi:10.3201/eid0702.010239. 11294736 PMC2631735

[40] Hopkins KL, Davies RH, Threlfall EJ. 2005. Mechanisms of quinolone resistance in *Escherichia coli* and *Salmonella*: recent developments. Int J Antimicrob Agents 25:358–373. doi:10.1016/j.ijantimicag.2005.02.006. 15848289

[41] Piddock LJ, Ricci V, Pumbwe L, Everett MJ, Griggs DJ. 2003. Fluoroquinolone resistance in *Campylobacter* species from man and animals: detection of mutations in topoisomerase genes. J Antimicrob Chemother 51:19–26. doi:10.1093/jac/dkg033. 12493783

[42] Luo N, Sahin O, Lin J, Michel LO, Zhang Q. 2003. *In vivo* selection of *Campylobacter* isolates with high levels of fluoroquinolone resistance associated with *gyrA* mutations and the function of the CmeABC efflux pump. Antimicrob Agents Chemother 47:390–394. doi:10.1128/AAC.47.1.390-394.2003. 12499221 PMC148968

[43] Dai L, Sahin O, Grover M, Zhang Q. 2020. New and alternative strategies for the prevention, control, and treatment of antibiotic-resistant *Campylobacter*. Transl Res 223:76–88. doi:10.1016/j.trsl.2020.04.009. 32438073 PMC7423705

[44] FDA. 2005. Enrofloxacin for poultry: withdrawal of approval of Bayer Corporation’s new animal drug application (NADA) 140–828 (Baytril). https://www.fda.gov/animal-veterinary/recalls-withdrawals/withdrawal-enrofloxacin-poultry.

[45] Price LB, Lackey LG, Vailes R, Silbergeld E. 2007. The persistence of fluoroquinolone-resistant *Campylobacter* in poultry production. Environ Health Perspect 115:1035–1039. doi:10.1289/ehp.10050. 17637919 PMC1913601

[46] Luo N, Pereira S, Sahin O, Lin J, Huang S, Michel L, Zhang Q. 2005. Enhanced in vivo fitness of fluoroquinolone-resistant *Campylobacter jejuni* in the absence of antibiotic selection pressure. Proc Natl Acad Sci USA 102:541–546. doi:10.1073/pnas.0408966102. 15634738 PMC545549

[47] Kirchhelle C. 2018. Pharming animals: a global history of antibiotics in food production (1935–2017). Palgrave Commun 4:96. doi:10.1057/s41599-018-0152-2.

[48] Engberg J, Aarestrup FM, Taylor DE, Gerner-Smidt P, Nachamkin I. 2001. Quinolone and macrolide resistance in *Campylobacter jejuni* and *C. coli*: resistance mechanisms and trends in human isolates. Emerg Infect Dis 7:24–34. doi:10.3201/eid0701.010104. 11266291 PMC2631682

[49] Desmonts M-H, Dufour-Gesbert F, Avrain L, Kempf I. 2004. Antimicrobial resistance in *Campylobacter* strains isolated from French broilers before and after antimicrobial growth promoter bans. J Antimicrob Chemother 54:1025–1030. doi:10.1093/jac/dkh473. 15537699

[50] Abraham S, Sahibzada S, Hewson K, Laird T, Abraham R, Pavic A, Truswell A, Lee T, O’Dea M, Jordan D, Elkins CA. 2020. Emergence of fluoroquinolone-resistant *Campylobacter jejuni* and *Campylobacter coli* among Australian chickens in the absence of fluoroquinolone use. Appl Environ Microbiol 86:e02765-19. doi:10.1128/AEM.02765-19. 32033955 PMC7117913

[51] Kim J, Park H, Kim J, Kim JH, Jung JI, Cho S, Ryu S, Jeon B. 2019. Comparative analysis of aerotolerance, antibiotic resistance, and virulence gene prevalence in *Campylobacter jejuni* isolates from retail raw chicken and duck meat in South Korea. Microorganisms 7:433. doi:10.3390/microorganisms7100433. 31658662 PMC6843641

[52] Daghrir R, Drogui P. 2013. Tetracycline antibiotics in the environment: a review. Environ Chem Lett 11:209–227. doi:10.1007/s10311-013-0404-8.

[53] Van Boeckel TP, Brower C, Gilbert M, Grenfell BT, Levin SA, Robinson TP, Teillant A, Laxminarayan R. 2015. Global trends in antimicrobial use in food animals. Proc Natl Acad Sci USA 112:5649–5654. doi:10.1073/pnas.1503141112. 25792457 PMC4426470

[54] Oh E, Jeon B. 2014. Role of alkyl hydroperoxide reductase (AhpC) in the biofilm formation of *Campylobacter jejuni*. PLoS One 9:e87312. doi:10.1371/journal.pone.0087312. 24498070 PMC3909096

[55] Hwang S, Ryu S, Jeon B. 2013. Roles of the superoxide dismutase SodB and the catalase KatA in the antibiotic resistance of *Campylobacter jejuni*. J Antibiot (Tokyo) 66:351–353. doi:10.1038/ja.2013.20. 23549350

[56] Kim M, Hwang S, Ryu S, Jeon B. 2011. Regulation of *perR* expression by iron and PerR in *Campylobacter jejuni*. J Bacteriol 193:6171–6178. doi:10.1128/JB.05493-11. 21908670 PMC3209207

[57] Jeon B, Zhang Q. 2007. Cj0011c, a periplasmic single-and double-stranded DNA-binding protein, contributes to natural transformation in *Campylobacter jejuni*. J Bacteriol 189:7399–7407. doi:10.1128/JB.01012-07. 17693521 PMC2168429

[58] McDermott PF, Bodeis-Jones SM, Fritsche TR, Jones RN, Walker RD. 2005. Broth microdilution susceptibility testing of *Campylobacter jejuni* and the determination of quality control ranges for fourteen antimicrobial agents. J Clin Microbiol 43:6136–6138. doi:10.1128/JCM.43.12.6136-6138.2005. 16333113 PMC1317209

