# Supplementary material for: Development of Fluoroquinolone Resistance through Antibiotic Tolerance in Campylobacter jejuni
Source: Microbiol Spectr. 2022 Sep 6;10(5):e01667-22. doi: 10.1128/spectrum.01667-22 (PMC9602944; doi:10.1128/spectrum.01667-22)
Supplement: Supplemental file 1 — Fig. S1 to S3. Download spectrum.01667-22-s0001.pdf, PDF file, 0.2 MB [file spectrum.01667-22-s0001.pdf]

SUPPLEMENTAL DATA

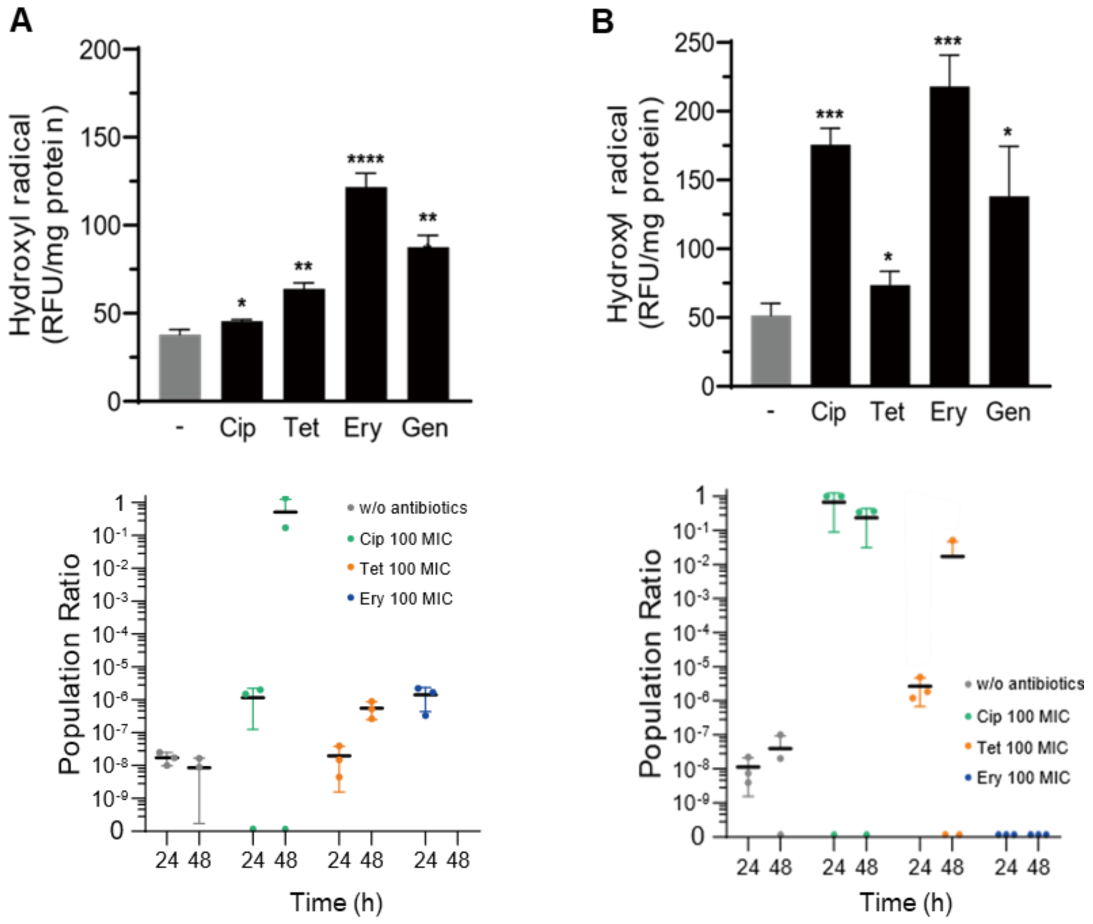

Fig. S1. Hydroxyl peroxide production by antibiotic treatment, and the development of fluoroquinolone (FQ) resistance in *C. jejuni* ATCC 33291 (A) and *C. jejuni* ATCC 33560 (B). Hydroxyl radical levels were measured after exposure to 100x MIC of ciprofloxacin (Cip), tetracycline (Tet), erythromycin (Ery), and gentamicin (Gen) for 24 hours. The experiments were repeated three times. Statistical analyses were conducted using the Student's *t*-test in comparison with the non-treated control (-). *: $P<0.05$, **: $P<0.01$, ***: $P<0.001$, and ****: $P<0.0001$

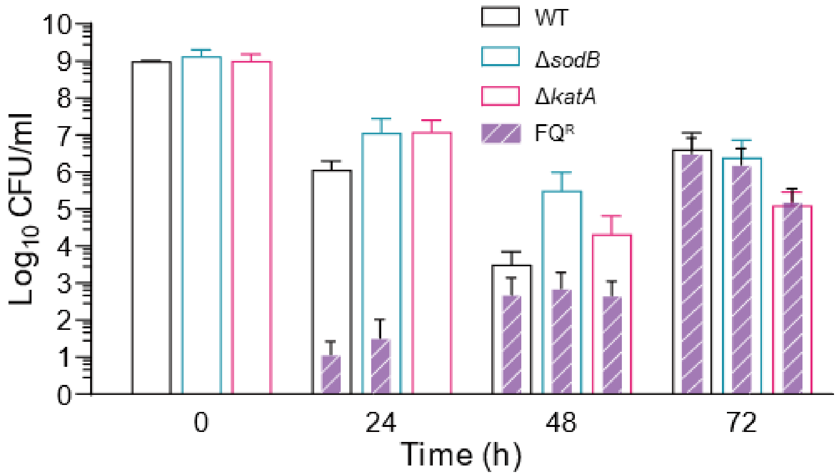

Fig. S2. Fluoroquinolone (FQ) resistance development in Δ*katA* and Δ*sodB* mutants in the presence of 100x MIC of ciprofloxacin. The results show the means and standard deviations of the levels of total *C. jejuni* (empty bars) and FQ^R *C. jejuni* (patterned fills) of the results from three independent experiments.

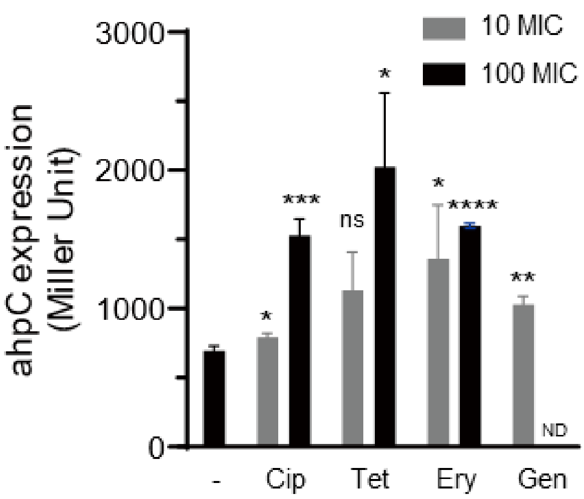

Fig. S3. Increased transcriptional level of *ahpC* during exposure to high concentrations of antibiotics. *C. jejuni* was exposed to 10x or 100x MIC of antibiotics for 24 hours before β-galactosidase assays. The assay was conducted as described previously (1). The results show the means and standard deviations of one representative experiment with three samples. The assay was repeated three times and produced similar results. Statistical analysis was conducted with Student's *t*-test. ns: non-significant, *: $P<0.05$, **: $P<0.01$, ***: $P<0.001$, and ****: $P<0.0001$.

Reference

1. Kim JC, Oh E, Hwang S, Ryu S, Jeon B. 2015. Non-selective regulation of peroxide and superoxide resistance genes by PerR in *Campylobacter jejuni*. *Front Microbiol*. 6:126.
